# Force oscillations underlying precision grip in humans with lesioned corticospinal tracts

**DOI:** 10.1016/j.nicl.2023.103398

**Published:** 2023-04-11

**Authors:** Charley W. Lafe, Fang Liu, Tyler W. Simpson, Chan Hong Moon, Jennifer L. Collinger, George F. Wittenberg, Michael A. Urbin

**Affiliations:** a Human Engineering Research Laboratories, VA RR&D Center of Excellence, VA Pittsburgh Healthcare System, Pittsburgh, PA 15206, USA; b Rehabilitation Neural Engineering Laboratories, University of Pittsburgh, Pittsburgh, PA 15213, USA; c Department of Physical Medicine & Rehabilitation, University of Pittsburgh, Pittsburgh, PA 15213, USA; d Department of Radiology, University of Pittsburgh, Pittsburgh, PA 15213, USA; f Department of Bioengineering, University of Pittsburgh, Pittsburgh, PA 15213, USA; e Department of Neurology, University of Pittsburgh, Pittsburgh, PA 15213, USA

**Keywords:** Precision grip, Motor control, Stroke, Corticospinal tract, White matter

## Abstract

Stability of precision grip depends on the ability to regulate forces applied by the digits. Increased frequency composition and temporal irregularity of oscillations in the force signal are associated with enhanced force stability, which is thought to result from increased voluntary drive along the corticospinal tract (CST). There is limited knowledge of how these oscillations in force output are regulated in the context of dexterous hand movements like precision grip, which are often impaired by CST damage due to stroke. The extent of residual CST volume descending from primary motor cortex may help explain the ability to modulate force oscillations at higher frequencies. Here, stroke survivors with longstanding hand impairment (n = 17) and neurologically-intact controls (n = 14) performed a precision grip task requiring dynamic and isometric muscle contractions to scale and stabilize forces exerted on a sensor by the index finger and thumb. Diffusion spectrum imaging was used to quantify total white matter volume within the residual and intact CSTs of stroke survivors (n = 12) and CSTs of controls (n = 14). White matter volumes within the infarct region and an analogous portion of overlap with the CST, mirrored onto the intact side, were also quantified in stroke survivors. We found reduced ability to stabilize force and more restricted frequency ranges in force oscillations of stroke survivors relative to controls; though, more broadband, irregular output was strongly related to force-stabilizing ability in both groups. The frequency composition and temporal irregularity of force oscillations observed in stroke survivors did not correlate with maximal precision grip force, suggesting that it is not directly related to impaired force-generating capacity. The ratio of residual to intact CST volumes contained within infarct and mirrored compartments was associated with more broadband, irregular force oscillations in stroke survivors. Our findings provide insight into granular aspects of dexterity altered by corticospinal damage and supply preliminary evidence to support that the ability to modulate force oscillations at higher frequencies is explained, at least in part, by residual CST volume in stroke survivors.

## Highlights

• Control of finger force is fundamental to everyday tasks requiring manual dexterity.
• Force stability is compromised in humans with longstanding corticospinal tract lesions.
• Oscillations in the force signal are linked to stability but altered by tract lesions.
• Modulating oscillations at higher frequencies is related to residual tract volume.

## 1 Introduction

Grasping and manipulating objects between the tactile pads of the thumb and fingertips via precision grip is a motor behavior integral to human function but often impaired after damage to the corticospinal tract (CST) (Lawrence and Kuypers, 1968, Ward et al., 2007). Maintenance of precision grip requires dynamic and static muscle contractions to stabilize forces exerted on an object. Forces exerted by the digits on a pencil while writing or on a fork while feeding, for example, are continuously graded and held constant to control the grasped object. Corticomuscular coherence underlying finger movements has been shown to predominate in the gamma band (30–60 Hz) during dynamic contractions (Omlor et al., 2007) and in the beta band (13–30 Hz) during static contractions (Conway et al., 1995, Kristeva et al., 2007). Beta-range coherence in the macaque has been shown between forelimb muscle activity and local field potentials in the spinal cord during the grip (i.e., dynamic) phase and motor cortex during the hold (i.e., static) phase (Oya et al., 2020). Taken together, these findings support the idea of a phase-specific compartmentalization of sensorimotor feedback loops within the beta band. Irrespective of the precise manner in which descending tracts constrain dynamic and static aspects of limb control, how force output is regulated by humans to stabilize precision grip is not well understood.

When stabilizing limb movement, oscillations in the force signal tend to be dominated by frequencies <2 Hz and show predictable trajectories over time. Inclusion of faster frequency oscillations and increased irregularity of the force time series is thought to reflect the flexible, adaptive capacity of the nervous system (Stergiou and Decker, 2011). For example, there is a narrowing of the power spectrum with age and disease (Lipsitz and Goldberger, 1992, Vaillancourt and Newell, 2002) and a broadening with development (Deutsch and Newell, 2003) and learning (Studenka et al., 2014). Human stroke survivors with longstanding motor impairment exhibit reduced faster-frequency contributions to paretic, upper (Lodha et al., 2013) and lower (Chow and Stokic, 2013) limb movements. Similar findings have been reported within the first month after stroke onset, with longitudinal analyses revealing improvements in the ability to stabilize force in relation to mean and target force but negligible recovery in the frequency content of force output (Chow and Stokic, 2014). The ability to stabilize force has been attributed to force oscillations <1 Hz (Fox et al., 2013, Moon et al., 2014, Lodha and Christou, 2017), and a shift toward higher frequencies albeit below 1 Hz are thought to enhance control (Fox et al., 2013, Lodha et al., 2013). Existing work on upper limb force output in stroke survivors has focused predominantly on force oscillations <1 Hz (Lodha et al., 2013, Kang and Cauraugh, 2015), but the functional relevance of faster frequency contributions to force stability is less well understood.

Stroke survivors retain the ability to modulate overall grip force within a limited force range (Lindberg et al., 2012), and it has been shown that both force generation and inhibition contribute to recovery of dexterous hand use (Plantin et al., 2022). Movement dysfunction after stroke is largely a consequence of the abnormal interaction between upper and lower motor neurons (Urbin et al., 2021), and it is thought that factors influencing voluntary drive along the CST to lower motor neurons alter the ability to stabilize muscle force (Farina and Negro, 2015). Microstructural integrity of the CST is an established prognostic indicator of motor recovery after stroke (Puig et al., 2013) and also predicts gains from therapy at the chronic stage (Lindenberg et al., 2012). At the chronic stage, however, atrophy of brain parenchyma lesioned by the stroke has evolved to the point where a significant loss of white matter volume is possible and, in turn, likely to have an adverse effect on limb control. To this end, the proportion of the CST overlap with the infarct region (i.e., lesion load) predicts the ability to coordinate dynamic finger forces 6 months following stroke onset (Pennati et al., 2020). However, it is unknown whether changes in residual CST volume determined by individual tract trajectories are linked to the ability to modulate force oscillations at higher frequencies.

Here, we sought to address three questions: First, how is the frequency composition and temporal irregularity of force output underlying precision grip altered by CST damage due to stroke? Second, how does the frequency composition and temporal irregularity of force output relate to force-stabilizing ability? Third, is the ability to modulate force oscillations at higher frequencies explained by the relative proportion of CST volume that remains in stroke survivors with longstanding lesions? To address these questions, we analyzed force signals recorded while individuals with hand impairment secondary to stroke and neurologically-intact controls performed a precision grip task that required dynamic scaling of motor output to stabilize low-level forces. Metrics were computed to quantify force oscillations in frequency and time domains, and diffusion spectrum imaging (DSI) was used to quantify white matter volume within the CST. Our findings reveal more restricted frequency ranges and reduced temporal irregularity in the force output of human stroke survivors that is tied to force-stabilizing ability but not related to impaired force-generating capacity. We found that stroke survivors’ ability to modulate force output at higher frequencies is associated with an asymmetry in the proportional volume of white matter contained within infarct and mirrored regions of residual and intact CSTs.

## 2 Materials and methods

### 2.1 Subjects

Seventeen stroke survivors with longstanding motor impairment due to stroke (10.2 ± 6.2 years post stroke, n = 7 female) and 14 neurologically-intact controls (n = 8 female, n = 13 right-hand dominant) participated in the study. One left-side dominant control was included to account for the ∼10 % prevalence of left-side dominance in the world population. All subjects provided signed informed consent to undergo research procedures approved by the Institutional Review Board at VA Pittsburgh Healthcare System in accordance with guidelines established by the Declaration of Helsinki. All but one stroke survivor, who sustained a hemorrhage, experienced ischemic strokes that resulted in a wide range of hand impairments (Table 1). Manual muscle testing (0–10 scale) of the paretic dorsal interosseous muscle into index finger abduction was used to evaluate strength (i.e., impairment) for descriptive purposes (Mendell and Florence, 1990).

**Table 1:** Clinical characteristic of stroke survivors. (R = right; L = left; ND = non-dominant; D = dominant; MCA = middle cerebral artery; VAO = vertebral artery occlusion; # = hemorrhagic stroke; Proportional Volume (%) = white matter volume within infarct region relative to total CST volume.)

**ID**	**Age**	**Sex**	**Stroke Type**	**Chronicity (months)**	**Pinch Force (N)**	**MMT**	**Side Affected**	**Proportional Volume (%)**
1	59	M	R MCA	120	34.2	T	ND (L)	30.9
2	58	M	R Basal Ganglia	117	26.3	7	ND (L)	38.7
3	60	M	R Unknown	201	17.7	T	ND (L)	–
4	71	F	R MCA	260	50.4	10	ND (L)	60.2
5	70	M	L Capsule	45	9.3	T	D (R)	81.7
6	61	F	L Capsule	55	19.1	6	D (R)	20.5
7	60	M	L Cerebellar	104	51.5	7	D (R)	–
8	72	F	L MCA	50	26.0	5	ND (R)	49.9
9	60	F	L Pontine	55	16.2	1	D (R)	–
10	74	M	L Basal Ganglia^#^	117	13.3	4	D (R)	–
11	66	F	R Capsule	197	23.9	9	D (L)	20.7
12	49	M	L MCA	221	16.4	1	D (R)	46.9
13	52	F	R MCA	38	11.3	T	ND (L)	65.7
14	47	F	L MCA	64	25.3	1	ND (R)	45.7
15	70	M	R MCA	76	11.8	T	ND (L)	–
16	72	M	R MCA	132	2.4	1	D (L)	57.2
17	74	M	L MCA	240	53.2	10	D (R)	60.6

MMT scale: 0 = no contractions felt in muscle; T = feeble contraction felt in the muscle with no visible movement of the segment; 1 = movement in horizontal plane through partial range of motion; 2 = movement in horizontal plane through complete range of motion; 3 = movement against gravity through partial range of motion; 4 = movement against gravity in test position with gradual release; and 5 = movement against gravity and test position maintained. Higher scores (6–10) reflect movement against gravity and maintenance of test position in the presence of slight pressure (6), slight to moderate pressure (7), moderate pressure (8), moderate-to-strong pressure (9), and strong pressure (10).

### 2.2 Experimental setup & task paradigm

**Fig. 1:**
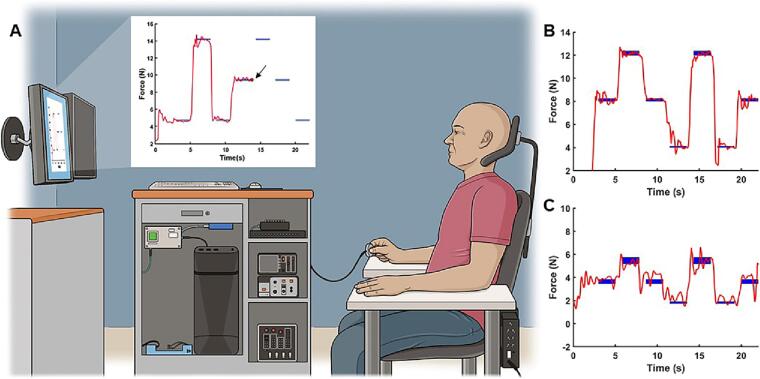
A) Experimental set up and representative force traces from a B) control and C) stroke subject. The solid red line represents the real-time force trace and blue rectangles reflect targets at each target force level. Note that only the targets and red circular cursor (black arrow in A) representing the current force output were visible to the subject. (For interpretation of the references to colour in this figure legend, the reader is referred to the web version of this article.)

Subjects sat in a chair approximately 1.16 m in front of a computer monitor (3840 horizontal pixel × 2160 vertical pixel resolution, 144 Hz refresh rate). Force signals were sampled at 200 Hz using a 6-axis force sensor (Mini40, ATI Industrial Automation). The pixel-to-Newton ratio was set at 64 pixels/N such that 64 pixels illuminated for every Newton of force applied along the z-axis of the sensor. Subjects held the sensor between the index finger and thumb with remaining digits fully flexed, increasing or decreasing the amount of force applied to control a circular red cursor (10-pixel diameter) in an upward or downward direction, respectively, as it moved across the display window (Fig. 1A). Some stroke survivors were unable to hold the sensor between the tactile pads of the index finger and thumb due to their impairments and, instead, adopted a key grip wherein the sensor was held between the side of the index finger and thumb. Photos were taken to ensure the specific hand posture adopted by an individual subject remained constant. A visuomotor task paradigm similar to those used in previous studies of motor skill learning (Reis et al., 2009) was created in MATLAB version 2021a (The MathWorks, Inc), which required dynamic and static contractions of intrinsic hand muscles to align the path of the moving red circle through rectangular targets representing 7, 14, and 21 % of maximum precision grip force (Fig. 1B and 1C). In this way, the task required the ability to scale and stabilize low-level forces used during precision grip via dynamic and static contractions, respectively.

A calibration procedure was administered at the start of the experiment to obtain measurements for establishing target templates and to define the noise floor of the sensor while it was positioned on a stable surface. First, maximal precision grip force was measured. Subjects were verbally exhorted by the experimenter to produce a true maximum voluntary contraction (MVC) used to quantify subjects’ force-generating capacity (Ikai and Steinhaus, 1961). Precision grip MVC was taken as the mean value calculated from 2 s centered around peak force. Next, a force-matching task at 10, 20, 30, 40, and 50 % of the MVC previously recorded was performed, which required subjects to stabilize precision grip at each force level on separate trials. Force signals during the MVC and force-matching task were recorded over 5 s. After a ∼ 1-min rest period, the calibration procedure was repeated a second time. These measurements were used to set target height and vertical target width. Target heights were set at 7, 14, and 21 % of MVC to approximate the low-level forces used during precision grip and also to ensure that the maximum difference between lower and upper force targets for subjects with large maximal precision grip forces did not exceed viewing limits on the display window. Vertical width of each respective target force was set by regressing variability in the force signal (i.e., 1 standard deviation) from the final 2 s of recordings onto each of the 5 force levels matched, with the intercept constrained to 0.

Three sets of 6 trials (18 total trials) were obtained from each subject. With each set of trials occurring on a separate day, minimizing the potential for fatigue. A total of 7 target forces were presented simultaneously at the start of a trial, at which time the red circle moved across the display window from left to right at a constant speed, resulting in a 22-s data sweep. A total of 6 consecutive trials were performed, but the circle idled at the left edge of the display window until a minimal force was applied to ensure that the subject was prepared and grip was maintained. The first target force in the series of 7 for a given trial was positioned 3 s into the sweep, allowing the subject adequate time to ramp force to its height. The inter-target interval between each set of adjacent targets (0.833 s) was a function of trial length (22 s), horizontal target length (2 s), and time of the initial target (3 s). This configuration was selected to approximate natural, dexterous actions that require dynamic transitions between stable states. The order of target force levels was pseudorandomized to ensure that *a)* each target force was preceded by a different target force, resulting in 6 possible force level-to-force level transitions, with each transition occurring only once within a trial, *b)* trials began and ended at the same force level to result in 3 target crossings at a single force level and 2 target crossings at each of the other force levels, and *c)* the initial target force level changed systematically such that each force level was presented as the initial target only once in a series of 3 trials. The initial target of each trial was excluded from analyses, resulting in a total of 36 target crossings obtained at each force level (2 target crossing per force per trial, 18 total trials).

### 2.3 Magnetic resonance imaging (MRI) & diffusion spectrum imaging (DSI)

A subset of stroke survivors (n = 12) in whom MRI was not contraindicated and all controls (n = 14) underwent MRI using a Prisma 3T scanner (Siemens, Erlangen, Germany) with 64-channel head/neck receiver radio-frequency (RF) coil. Structural MRI was acquired using T1 magnetization-prepared rapid acquisition with gradient-echo (MPRAGE) and T2 sampling perfection with application-optimized contrasts using a different flip angle evolutions (SPACE) sequence with the following parameters: repletion time (TR)/inversion time (TI)/echo time (TE) = 1900/900/1.67 ms, voxel resolution = 1.3-mm isotropic, acceleration factor = 2, acquisition time = 2 min 30 s; and TR/TE = 3200/412 ms, voxel resolution = 1-mm isotropic, acceleration factor = 2, acquisition time = 3 min 49 s, respectively). DSI data were acquired via a single-shot, twice-refocused, two-dimensional, multi-slice spin echo planar imaging (*EPI*) sequence (TR/TE = 2480/99.2 ms, voxel resolution = 2-mm isotropic, multi-band factor = 4, partial Fourier factor in phase encoding (PE) = 6/8, 258 diffusion directions with *b*-values of 4000 s/mm^2^ and one with a *b*-value of 0, echo spacing = 0.69 ms, and PE in anterior-to-posterior direction; total acquisition time = 11 min). A separate sequence with a *b*-value of 0 was acquired (posterior-to-anterior PE direction, acquisition time = 19 s) to correct for spatial distortion in the diffusion weighted MR image.

### 2.4 Data processing: Force stability and force output

Force signals were analyzed using MATLAB version 2021a. Signals from each target crossing (2-s time series, 400 samples) were de-trended and filtered using a 4th order Butterworth filter with a 12 Hz cutoff. Data from an individual target crossing were removed if absolute error was > 3 SD from the mean of a given subject’s distribution at each respective target force level. Absolute error was calculated as |Σ(Τ − *f*_i_)|, where Τ is the target force level, and *f*_i_ is the *i*^th^ force sample.

Force stability was quantified using both coefficient of variation (*CV*) and root-mean-square error (*RMSE*) to characterize variability around the mean force and target force, respectively. *RMSE* was calculated as [Σ(Τ − *f*_i_)^2^/n – 1]^1/2^, where n is the total number of force samples.

Force output *in the frequency domain* was calculated using Welch’s spectral power density estimate method via the ‘*pwelch*’ function in MATLAB. Slope of the log-power vs log-frequency regression was quantified (Lipsitz, 1995). Spectral slope (*Slope*) provides a global measure of force output in the frequency domain. As noted previously, most prior studies have focused on the relative contribution of frequencies <1 Hz with fewer including frequency bands >1 Hz (e.g., Slifkin and Newell, 2000, Chow and Stokic, 2014, Moon et al., 2014). This approach assumes a homogenous frequency range containing most power in the signal across subjects. To account for inter-subject variation, we also quantified the frequency limit that accounted for 95 % of the total power (*F95*) in the signal.

Force output *in the time domain* was quantified using fuzzy measure entropy (*FuzMEn*) (Liu and Zhao, 2011, Liu et al., 2013). *FuzMEn* was chosen over other entropy metrics (e.g., approximate entropy, sample entropy, and fuzzy entropy) because it considers both global and local characteristics of biological signals and has been shown to perform better with shorter time series (Chen et al., 2009, Liu et al., 2018). The similarity weight, *n*, and tolerance threshold, *r*, of the exponential defining the fuzzy membership function for both local and global properties were set to 2 and 0.15, respectively (Lin et al., 2017, John et al., 2022).

### 2.5 Data processing: diffusion spectrum imaging

**Fig. 2:**
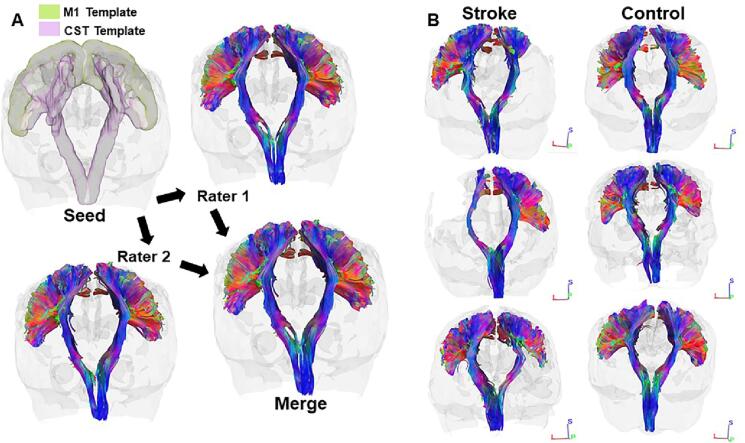
A) Independent raters manually segmented fibers in DSI Studio. M1 and CST Templates were used as separate regions of interest. Fibers were segmented after seeding in each region of interest, resulting in four segmentations for a given subject per rater. The resulting fibers tracts from Rater 1 and Rater 2 were merged to generate a final tract from which white matter volumes were quantified. B) Representative CSTs from three stroke survivors (subject IDs 4, 14, and 16 from Table 1) and three control subjects. Note the differential loss of white matter among stroke survivors.

DSI data preprocessing was completed using FSL (https://www.fmrib.ox.ac.uk/fsl) and included motion correction, eddy correction, as well as co-registration between T1, T2, MNI152 standard brain images and the DSI image. Stroke lesion masks were hand drawn based on T1 and T2, then co-registered to the DSI image. DSI, an extension of diffusion tensor imaging, enables quantification of anisotropic diffusion of water molecules to reconstruct white matter tracts. DSI Studio software (https://dsi-studio.labsolver.org) was used to visualize residual and intact CSTs in stroke survivors and for both CSTs of controls. A deterministic fiber tracking algorithm (angular threshold = 60°, step size = 1 mm) was used to reconstruct CSTs in both hemispheres from primary motor cortex (i.e., precentral gyrus) to the caudal end of the image volume at the level of the brainstem based on subject-specific tract trajectories (Yeh et al., 2013). Fibers were generated using the co-registered precentral gyrus (i.e., primary motor cortex, M1) from the Harvard-Oxford Cortical Structural Atlas and the CST template from HCP842 Tractography Atlas as separate regions of interest (Fig. 2A). The precision grip task performed in this study involved extrinsic and intrinsic hand muscles. Given the lack of strict somatotopy in M1, (Schieber and Hibbard, 1993) as well as the potential for fiber loss and shifts in the location of the hand representation in individuals with longstanding stroke (Fig. 2B), our goal for combining fibers generated from separate regions of interest that covered a wide area of cortex was to ensure all residual fibers originating in primary motor cortex and belonging to the CST were included. This approach was necessary given that several stroke survivors in our small cohort showed significant loss of white matter at various locations along the length of the CST. Fibers generated by seeding in both regions of interest were segmented separately by two independent raters who sought to retain only those fibers originating within and descending from primary motor cortex. Tracts segmented by both raters were merged prior to quantifying white matter volume (mm^3^) within each CST (i.e., *total volume*) (CST corresponding to dominant and non-dominant hand in controls; residual and intact CST in stroke survivors). White matter volumes within the infarct region of the residual CST and an analogous region of overlap, mirrored onto the intact CST, were quantified to derive *compartment volume*. Volumes within either region were normalized to the entire volume in each respective CST to derive *proportional volume*. The ratio of *proportional volumes* between the residual and intact CSTs was used to derive *proportional volume asymmetry*.

### 2.6 Statistical analyses

Since approximately half of the stroke sample (8/17 subjects) was affected on the non-dominant side, force data used in analyses were acquired from the non-dominant side in half of controls (7/14 subjects) and from the dominant side in the other half (7/14 subjects). Independent samples t-tests were used to test for a difference in age and maximal precision grip force between stroke and control groups. All metrics of force stability and force output were submitted to a 2 (group) × 3 (target force level) mixed-ANOVA. Normality and homogeneity of variance assumptions were verified through visual inspection of Q-Q plots and Mauchly’s test of sphericity, respectively. Greenhouse-Geisser correction statistics were used when sphericity could not be assumed. The Bonferroni test was used to correct for multiple comparisons. Statistical tests were computed using SPSS version 23 (SPSS Inc, Chicago), and significance was set at *p* < 0.05 a priori.

A linear mixed-effects model was fit to assess the log-linear relation between metrics of force stability and force output. Models were fit using the ‘*fitlme*’ function in MatLab. Individual models were generated for both force stability metrics (*CV*, *RMSE*) using each force output metric in frequency (*Slope*, *F95*) and time (*FuzMEn*) domains as predictors at the subject level (level-1; fixed effects), resulting in 6 separate models. To avoid imaginary numbers, the log transform of the *Slope* metric was not used. A categorical predictor for group (stroke, control) was included in each model. We hypothesized that more broadband, temporally irregular oscillations in force output would be associated with the ability to stabilize force and were less interested in how force level would moderate this relation. We therefore did not include target force level as a predictor and assumed our hypothesis would hold across force levels. Metrics of force stability and force output were quantified at each individual target crossing, resulting in 108 data points for each subject (18 trials × 6 target crossings per trial). Random effects were grouped by subject (level-2; random effects), allowing slope and intercept to vary. A diagonal covariance matrix was used.

Pearson product-moment correlation was used to examine the correlation between precision grip MVC of the paretic hand and metrics of force stability (i.e., *CV*, *RMSE*) and force output (i.e., *Slope*, *F95*, and *FuzMEn*) at each target force level. Metrics were averaged over all 18 trials (2 target crossings per target force level per trial) at each target force level. Precision grip MVC, as opposed to scores from manual muscle testing, was entered into analyses because it is a valid and reliable measure of impairment (Mathiowetz et al., 1984, Mathiowetz et al., 1985) that provides a more expansive scale to capture deficits in the ability to activate muscle and also is specific to the task used in experiments.

Inter-rater reliability was established for fiber number within each CST (dominant and non-dominant CST in controls; residual and intact CST in stroke survivors). Intraclass Correlation Coefficient estimates and 95 % confidence intervals were calculated using SPSS version 23 based on absolute-agreement, two-way mixed-effects models. 2 (group) × 2 (CST) mixed-ANOVA was used to test for differences in *total volume* within each CST; paired-samples t-tests were used to test for differences in *compartment volume* and *proportional volume* in the stroke group. Normality and homogeneity of variance assumptions were verified through visual inspection of Q-Q plots and Mauchly’s test of sphericity, respectively. Greenhouse-Geisser correction statistics were used when sphericity could not be assumed. The Bonferroni test was used to correct for multiple comparisons, and significance was set at *p* < 0.05 a priori. Finally, Pearson product-moment correlation was used to examine the association between *FuzMEn* and *proportional volume asymmetry* at each target force level. Since higher FuzMEn implies a greater contribution of higher frequency components in the signal, as indicated by its high collinearity with *Slope* (*r* = 0.89) and *F95* (*r* = 0.94), we limited correlational analyses to *FuzMEn* to avoid redundancy. The *proportional volume asymmetry* metric was used in an effort to account for volumetric changes in the area of overlap with the CST while also accounting for possible upregulation of the contralesional, intact CST after stroke. The Benjamini-Hochberg method was used to control for the false discovery rate at the 0.05 level.

## 3 Results

There was no difference in age (*t*(28) = 0.115, *p* = 0.909) between control (M = 62.8 years, S.E. = 1.4 years) and stroke (M = 63.1 years, S.E. = 2.1 years) groups. Precision grip MVC was reduced (*t*(29) = 3.92, *p* < 0.001, *d* = 1.42) in the stroke group (M = 24.01 N, S.E. = 3.69 N) relative to the control group (M = 44.65 N, S.E. = 3.68 N), indicating that force-generating capacity was impaired in stroke survivors.

### 3.1 Force stability (CV, RMSE): control vs stroke

**Fig. 3:**
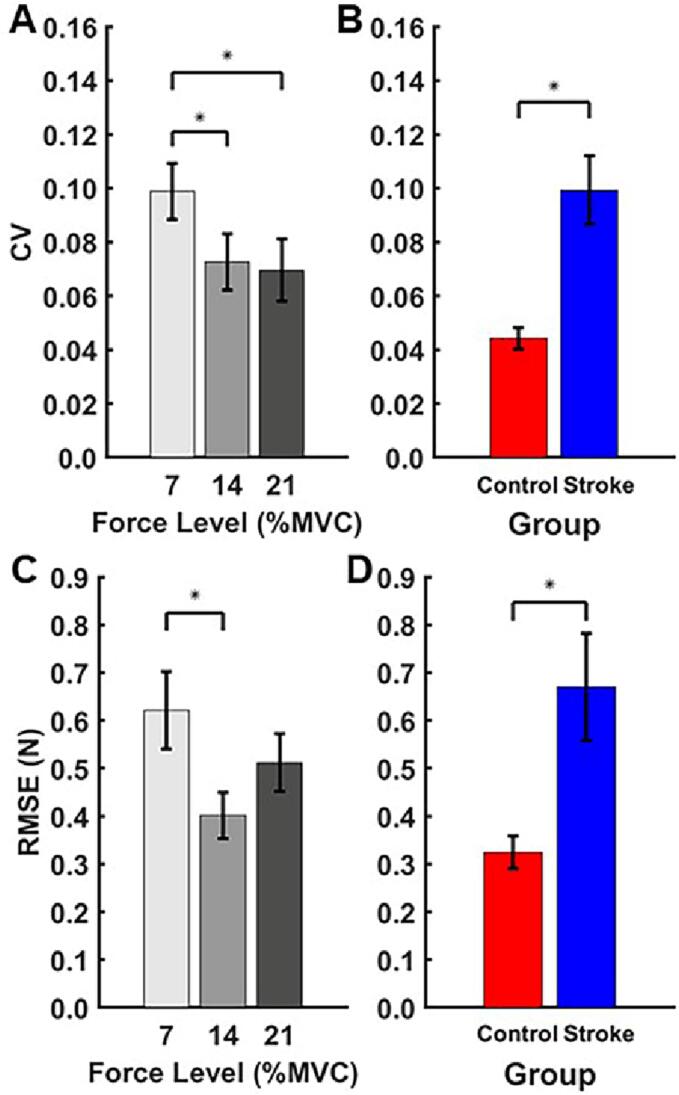
Force stability, as measured by *CV*, as a function of A) target force level and B) group. Force stability, as measured by *RMSE*, as a function of C) target force level and D) group. Error bars reflect standard error of the mean (*p < 0.05).

*CV*, which reflects variability about the mean force, as a function of target force level and group is shown in Fig. 3A and 3B, respectively. The mixed ANOVA revealed main effects of force level (F_(1.05,30.33)_ = 7.17, *p* = 0.011, $\eta_{p}^{2}$=0.20) and group (F_(1, 29)_ = 14.48, *p* = 0.001, $\eta_{p}^{2}$= 0.35). Post-hoc analysis revealed CV at the 7 % force level was significantly larger than at the 14 % force level (M_diff_ = 0.028, S.E. = 0.009, 95 %CI [0.005, 0.05], *p* = 0.01, *d* = 0.56) and 21 % force level (M_diff_ = 0.031, S.E. = 0.012, 95 %CI [0.009, 0.054], *p* = 0.003, *d* = 0.634). On average, the control group (M = 0.04, S.E. = 0.010) exhibited a lower *CV* than the stroke group (M = 0.1, S.E. = 0.01).

*RMSE*, which reflects variability about the target force, as a function of target force level and group is shown in Fig. 3C and 3D, respectively. The mixed ANOVA revealed main effects of force level (F_(1.2,34.88)_ = 6.5, *p* = 0.011, $\eta_{p}^{2}$=0.18) and group (F_(1, 29)_ = 7.91, *p* = 0.009, $\eta_{p}^{2}$= 0.21). Post-hoc analysis revealed RMSE was significantly higher at the 7 % force level (M_diff_ = 0.22 N, S.E. = 0.06 N, 95 %CI [0.068, 0.361], *p* = 0.002, *d* = 0.53) compared to the 14 % force level. On average, the control group (M = 0.31 N, S.E. = 0.1 N) exhibited lower *RMSE* than the stroke group (M = 0.67 N, S.E. = 0.09 N). In summary, stability around the mean force and target force were reduced in stroke survivors relative to controls.

### 3.2 Force output (slope, F95, FuzMEn): control vs stroke

**Fig. 4:**
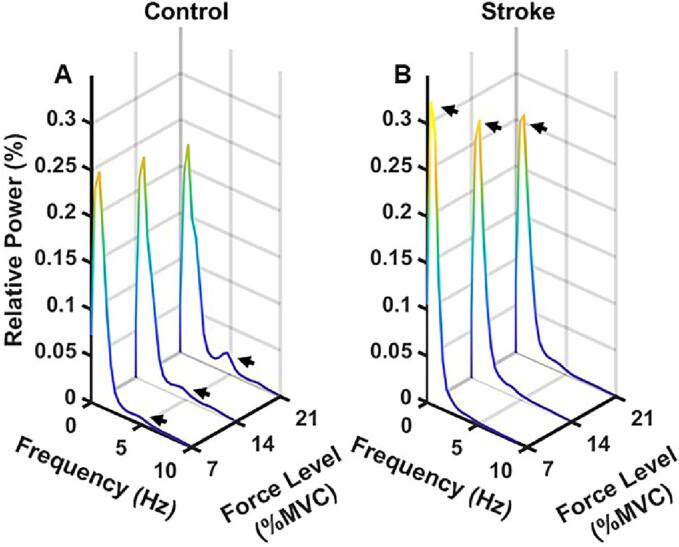
Relative spectral density as a function of force level for A) control and B) stroke groups. Each line represents the relative power at each force level averaged over all 36 target crossings (2 per force per trial, for 18 trials) across all subjects. Note the elevated relative power at the lower frequency peak (black arrows) in stroke survivors and second peak (black arrows) in controls.

Fig. 4A and 4B show spectral power at 7 %, 14 %, and 21 % target force levels in control and stroke groups, respectively. A greater slow-frequency contribution in stroke survivors and a noticeable increase in the power spectrum in controls, reflected by the second peak situated around 4–6 Hz, can be observed from these plots. Note that the second peak is absent in stroke survivors.

**Fig. 5:**
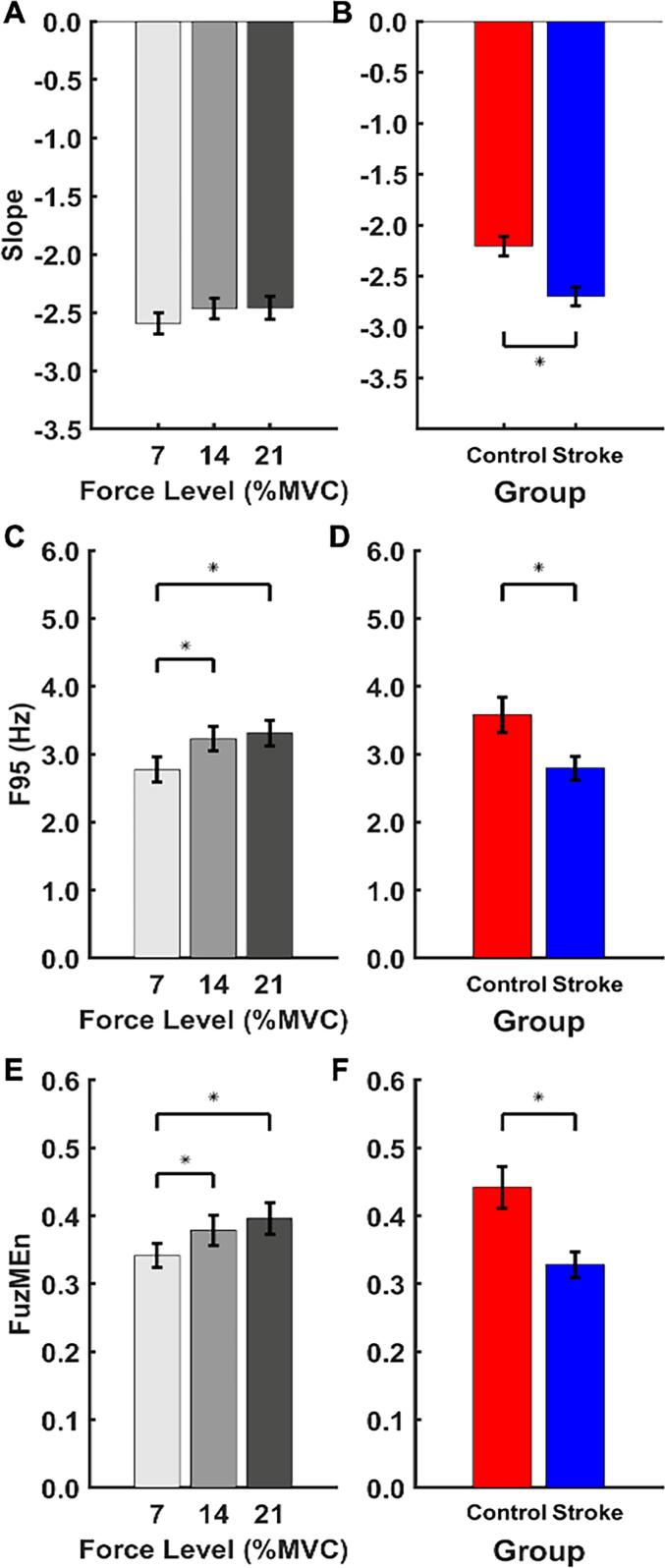
Force output in frequency (*Slope* and *F95*) and time (*FuzMEn*) domains. *Slope* as a function of A) force level and B) group is shown in the top panel. *F95* as a function of C) force level and D) group is shown in the middle panel. *FuzMEn* as a function of E) force level and F) group is shown in the bottom panel. Error bars reflect standard error of the mean. (*p < 0.05).

*Slope*, which is the slope of the log-power vs log-frequency regression and reflects force output in the frequency domain, as a function of target force level and group is shown in Fig. 5A and 5B, respectively. The mixed ANOVA revealed a main effect for group (F_(1, 29)_ = 13.87, *p* < 0.001, $\eta_{p}^{2}$= 0.32). On average, the control group (M = −2.20, S.E. = 0.1) showed a flatter, less negative *Slope* than the stroke group (M = −2.7, S.E. = 0.08). A less negative slope in the control group indicates an increase in faster-frequency contributions relative to the stroke group, which is consistent with spectral profiles presented in Fig. 4.

*F95*, which is the frequency limit accounting for 95 % of the total power in the force signal and also reflects force output in the frequency domain, *a*s a function of target force level and group is shown in Fig. 5C and 5D, respectively. The mixed ANOVA revealed main effects of force level (F_(1.68,48.89)_ = 9.63, *p* < 0.001, $\eta_{p}^{2}$= 0.25) and group (F_(1, 29)_ = 5.17, *p* = 0.031, $\eta_{p}^{2}$= 0.15). Post-hoc analysis revealed a significant increase in the frequency limit that accounted for 95 % of the power as a function of force level. When compared to the 7 % force level, there was a higher frequency limit at both the14% force level (M_diff_ = 0.46 Hz, S.E. = 0.12 Hz, 95 %CI [0.15 Hz, 0.77 Hz], p = 0.002, *d* = 0.47) and 21 % force level (M_diff_ = 0.49 Hz, S.E. = 0.15 Hz, 95 %CI [0.18 Hz, 0.81 Hz], p < 0.001, *d* = 0.5). On average, the control group (M = 3.54 Hz, S.E. = 0.24 Hz) exhibited higher *F95* than the stroke group (M = 2.8 Hz, S.E. = 0.19 Hz). Results pertaining to *Slope* and *F95* indicate that force oscillations in the frequency domain are altered by stroke and result in a more restricted frequency range.

*FuzMEn*, which reflects the predictability of the force signal time series, as a function of target force level and group is shown in Fig. 5E and 5F, respectively. The mixed ANOVA revealed main effects of force level (F_(1.39,40.34)_ = 12.51, *p* < 0.001, $\eta_{p}^{2}$= 0.30) and group (F_(1, 29)_ = 10.93, *p* = 0.003, $\eta_{p}^{2}$= 0.27). *FuzMEn* increased as a function of force level. Post-hoc analysis revealed a significant increase from 7 % to 14 % force levels (M_diff_ = 0.04, S.E. = 0.01, 95 %CI [0.009, 0.075], p = 0.007, *d* = 0.39) and from 7 % to 21 % force levels (M_diff_ = 0.07, S.E. = 0.02, 95 %CI [0.033, 0.099], p < 0.001, *d* = 0.61). On average, the control group (M = 0.45, S.E. = 0.05) showed higher *FuzMEn* than the stroke group (M = 0.33, S.E. = 0.02). Consistent with results from the frequency domain, force oscillations in the time domain are altered by stroke, resulting in reduced temporal irregularity (i.e., oscillations in the force signal are more predictable).

### 3.3 Force stability vs force output

**Fig. 6:**
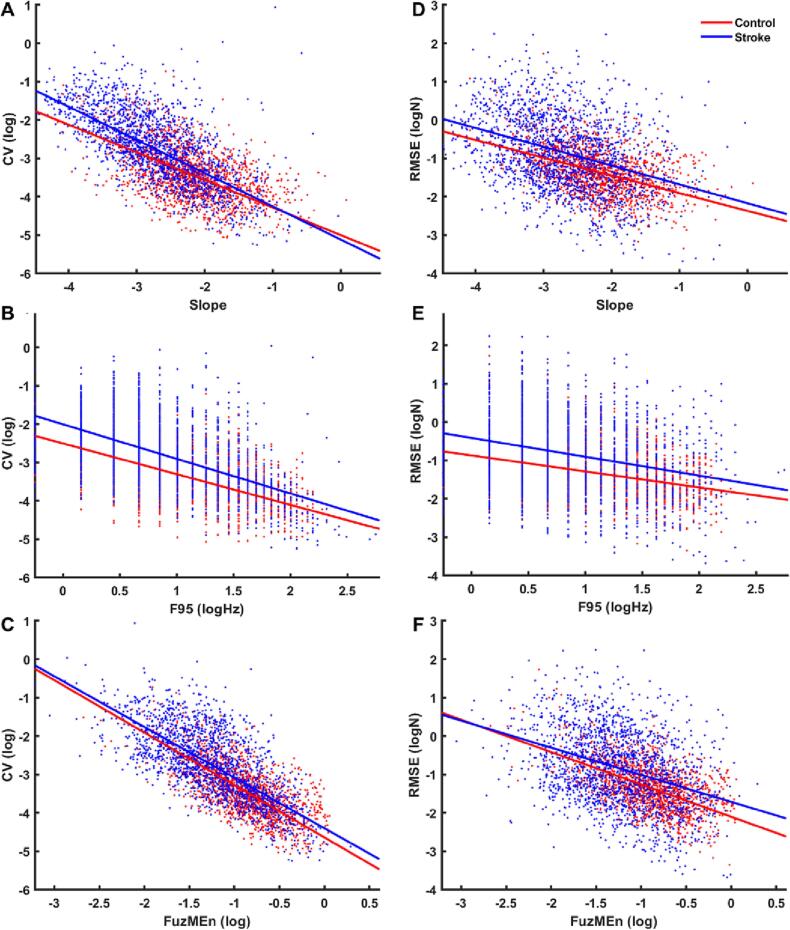
Scatter plots illustrating results of mixed effects models: force stability (*CV*, *RMSE*) vs force output in frequency and time domains (*Slope*, *F95*, *FuzMEn*). The left column contains fitted models for log-*CV* vs A) *Slope*, B) log-*F95* and C) log-*FuzMEn*. The right column contains fitted models for log-*RMSE* vs D) *Slope*, E) log-*F95* and F) log-*FuzMEn*. Red (control) and blue (stroke) data points correspond to individual target crossings (108 per subject). Red and blue lines reflect the predicted group averaged values for control and stroke groups, respectively. (For interpretation of the references to colour in this figure legend, the reader is referred to the web version of this article.)

The individual mixed-effects models revealed a significant negative relation between *CV* and *Slope* (F_(1,3344)_ = 215.3, *p* < 0.001, Fig. 6A), *F95* (F_(1,3344)_ = 155.44, *p* < 0.001, Fig. 6B) and *FuzMEn* (F_(1,3344)_ = 654.59, *p* < 0.001, Fig. 6C). There were negative coefficients for *Slope* (β = −0.72), *F95* (β = −0.8), and *FuzMEn* (β = −1.36). The same trends were found between *RMSE* and *Slope* (F_(1,3344)_ = 121.2, *p* < 0.001, Fig. 6D), *F95* (F_(1,3344)_ = 46.44, *p* < 0.001, Fig. 6E), and *FuzMEn* (F_(1,3344)_ = 177.88, *p* < 0.001, Fig. 6F). There were negative coefficients for *Slope* (β = −0.46), *F95* (β = −0.42), and *FuzMEn* (β = −0.84). In short, greater faster-frequency composition and temporal irregularity were associated with enhanced force stability. Additionally, there was a significant group × *Slope* (β = −0.14, *p* = 0.026) interaction term for the model predicting *CV*. Each model showed significant between-subject variance (level-2, random effects) for both slope (β) and intercept terms, with greater variability in the intercept than the slope, indicating that the relationship between force stability (i.e., *CV* and *RMSE*) and individual predictors was relatively consistent across subjects. The complete parameterization of models is reported in Table 2, Table 3, respectively.

**Table 2:** Linear mixed-effects models for *CV* vs *Slope*, *F95*, and *FuzMEn*.

**CV vs Slope:***Adj-R^2^* = 0.644					
Fixed effects					
	Estimate (±S.E.)	t-statistic	*p-*value	Lower	Upper
Constant	−4.997 (±0.155)	−32.365	<0.001	−5.303	−4.697
Stroke	−0.065 (±0.209)	−0.312	=0.755	−0.475	0.345
Slope	−0.719 (±0.049)	−14.673	<0.001	−0.815	−0.623
Slope*Stroke	−0.144 (±0.065)	−2.223	=0.026	−0.271	−0.017

Random effects					

	Estimate	Lower	Upper	Residual	
Constant	0.532	0.397	0.713	0.535	
Slope	0.154	0.111	0.213		
**CV vs F95:***Adj-R^2^* = 0.577					
Fixed Effects					
	Estimate (±S.E.)	t-statistic	*p-*value	Lower	Upper
Constant	−2.503 (±0.132)	−18.971	<0.001	−2.761	−2.244
Stroke	0.53 (±0.176)	3.012	=0.003	−0.185	0.875
F95	−0.801 (±0.064)	−12.468	<0.001	−0.927	−0.675
F95*Stroke	−0.092 (±0.085)	−1.078	=0.281	−0.26	0.075

Random effects					
	Estimate	Lower	Upper	Residual	
Constant	0.466	0.357	0.608	0.582	
F95	0.203	0.145	0.283		
**CV vs FuzMEn:***Adj-R^2^* = 0.739					
Fixed effects					
	Estimate (±S.E.)	t-statistic	*p-*value	Lower	Upper
Constant	−4.638 (±0.109)	−42.59	<0.001	−4.851	−4.424
Stroke	0.279 (±0.148)	1.892	=0.059	−0.01	0.569
FuzMEn	−1.364 (±0.053)	−25.59	<0.001	−1.469	−1.26
FuzMEn*Stroke	0.05 (±0.069)	0.723	*=*0.469	−0.086	0.187

Random effects					
	Estimate	Lower	Upper	Residual	
Constant	0.389	0.297	0.509	0.459	
FuzMEn	0.153	0.106	0.222

**Table 3:** Linear mixed-effects models for *RMSE* vs *Slope*, *F95*, and *FuzMEn*.

**RMSE vs Slope:***Adj-R^2^* = 0.525					
Fixed effects					
	Estimate (±S.E.)	t-statistic	*p-*value	Lower	Upper
Constant	−2.369 (±0.193)	−17.012	<0.001	−2.642	−2.096
Stroke	0.192 (±0.189)	1.02	=0.308	−0.177	0.561
Slope	−0.462 (±0.042)	−11.009	<0.001	−0.545	−0.379
Slope*Stroke	0.018 (±0.055)	−0.323	=0.747	−0.125	0.089

Random effects					
	Estimate	Lower	Upper	Residual	
Constant	0.457	0.34	0.616	0.605	
Slope	0.112	0.075	0.167		
**RMSE vs. F95:***Adj-R^2^* = 0.493					
Fixed Effects					
	Estimate (±S.E.)	t-statistic	*p-*value	Lower	Upper
Constant	−0.868 (±0.159)	−5.472	<0.001	−1.178	−0.557
Stroke	0.4 (±0.212)	1.886	=0.059	−0.016	0.816
F95	−0.417 (±0.061)	−6.815	<0.001	−0.537	−0.297
F95*Stroke	0.053 (±0.081)	−0.661	=0.508	−0.213	0.105

Random effects					
	Estimate	Lower	Upper	Residual	
Constant	0.568	0.437	0.737	0.626	
F95	0.185	0.129	0.263		
**RMSE vs FuzMEn:***Adj-R^2^* = 0.551					
Fixed effects					
	Estimate (±S.E.)	t-statistic	*p-*value	Lower	Upper
Constant	−2.1 (±0.126)	−16.641	<0.001	−2.347	−1.852
Stroke	0.373 (±0.171)	2.18	=0.029	0.037	0.708
FuzMEn	−0.842 (±0.063)	−13.337	<0.001	−0.965	−0.717
FuzMEn*Stroke	0.157 (±0.081)	1.916	*=*0.055	−0.004	0.318

Random effects					
	Estimate	Lower	Upper	Residual	
Constant	0.447	0.34	0.587	0.575	
FuzMEn	0.174	0.119	0.255		

### 3.4 Force-generating capacity vs force output in stroke survivors

There was no significant correlation between maximal grip force (MVC) and CV across target force levels [7 %: (*r* = 0.29, *p* = 0.262), 14 %: (*r* = −0.25, *p* = 0.335), 21 %: (*r* = −0.35, *p* = 0.175)] or RMSE at the 14 % (*r* = 0.37, *p* = 0.141) and 21 % (*r* = 0.42, *p* = 0.097) force levels. There was a significant positive correlation between RMSE and MVC at the 7 % (*r* = 0.6, *p* = 0.011) force level.

There was no significant correlation between maximal precision grip force (MVC) and any metric of force output in frequency or time domains across target force levels in the stroke group: *Slope* [7 %: (*r* = −0.21, *p* = 0.544), 14 %: (*r* = −0.001, *p* = 0.939), 21 %: (*r* = 0.26, *p* = 0.408)]; *F95* [7 %: (*r* = −0.14, *p* = 0.289), 14 %: (*r* = −0.09, *p* = 0.679), 21 %: (*r* = 0.17, *p* = 0.677)]; and *FuzMEn* [7 %: (*r* = −0.27, *p* = 0.237), 14 %: (*r* = −0.15, *p* = 0.787), 21 %: (*r* = 0.16, *p* = 0.802)]. The lack of any correlation across force levels suggests that the frequency composition and temporal irregularity of force output in stroke survivors is not due to impaired force-generating capacity.

### 3.5 CST white matter volume: control vs stroke

**Fig. 7:**
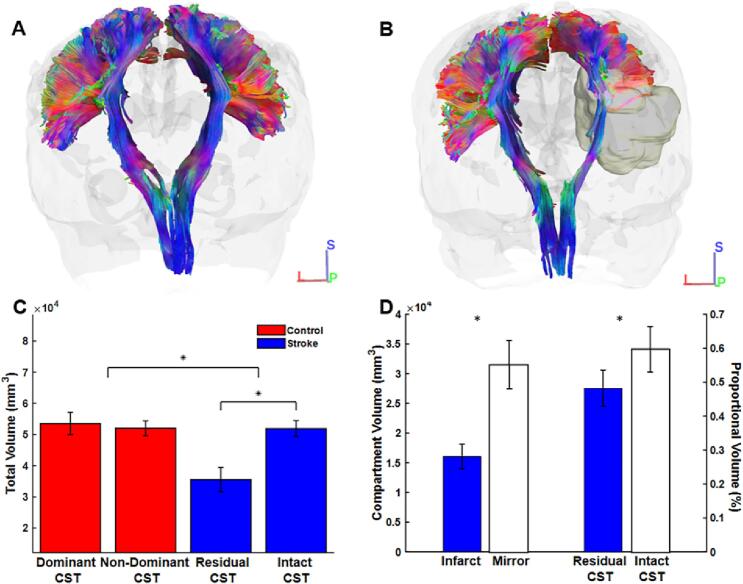
Coronal posterior view of CSTs in representative A) control and B) stroke subjects. The dominant CST is contained within the left hemisphere of the control subject, and the residual CST is contained within the right hemisphere of the stroke survivor (infarct compartment shown in yellow). Bar plots illustrating C) *total volume* by group (control vs stroke) and CST (dominant vs non-dominant vs residual vs intact); D) *compartment volume* (left vertical axis) and *proportional volume* (right vertical axis). (*p < 0.05). (For interpretation of the references to colour in this figure legend, the reader is referred to the web version of this article.)

Representative CSTs for a control subject and stroke survivor are presented in Fig. 7A and 7B, respectively. ICCs established a high degree of agreement between raters on fiber number in the control group [dominant CST: (ICC = 0.771, 95 % CI = 0.106 - 0.935); non-dominant CST: (ICC = 0.946, 95 % CI = 0.163 - 0.989)] and stroke group [residual CST: (ICC = 0.987, 95 % CI = 0.928 - 0.997); intact CST: (ICC = 0.986, 95 % CI = 0.951 - 0.996)]. *Total volume* as a function of group and CST (i.e., dominant and non-dominant CST in controls; residual and intact CST in stroke) is shown in Fig. 7C. The mixed ANOVA revealed main effects of group (F_(1, 24)_ = 13.46, *p* < 0.001, $\eta_{p}^{2}$=0.36) and CST (F_(1,24)_ = 8.92, *p* = 0.006, $\eta_{p}^{2}$= 0.27). There was also a significant group × CST interaction (F_(1, 24)_ = 12.88, *p* < 0.001, $\eta_{p}^{2}$=0.35). *Total volume* (mm^3^) was not significantly different between dominant and non-dominant CSTs (M_diff_ = 1496.0, S.E. = 3381.96, 95 %CI [-8227.46, 11219.46], *p* = 0.999, *d* = 0.17) in the control group. However, *total volume* of the residual CST was significantly lower than in the intact CST (M_diff_ = −16366.67, S.E. = 3652.94, 95 %CI [-26869.2, −5864.13], *p* < 0.001, *d* = −1.84). Overall, t*otal volume* was reduced in the residual CST compared to the intact CST and relative to both CSTs in controls.

Infarct and mirror *compartment volumes* (mm^3^), as well as residual and intact CST *proportional volumes* (%) are shown in Fig. 7D. Paired-samples t-tests revealed that white matter volume within the infarct compartment of the residual CST was significantly reduced relative to the mirror compartment of the intact CST (*t*_(11)_ = −3.43, *p* = 0.006, *d* = −0.99). Paired-samples t-tests also showed that *proportional volume* of the residual CST contained within the infarct compartment was significantly reduced relative to the *proportional volume* of the intact CST contained within the mirror compartment (*t*_(11)_ = −2.96, *p* = 0.013, *d* = 0.4).

### 3.6 CST white matter volume & force output

**Fig. 8:**
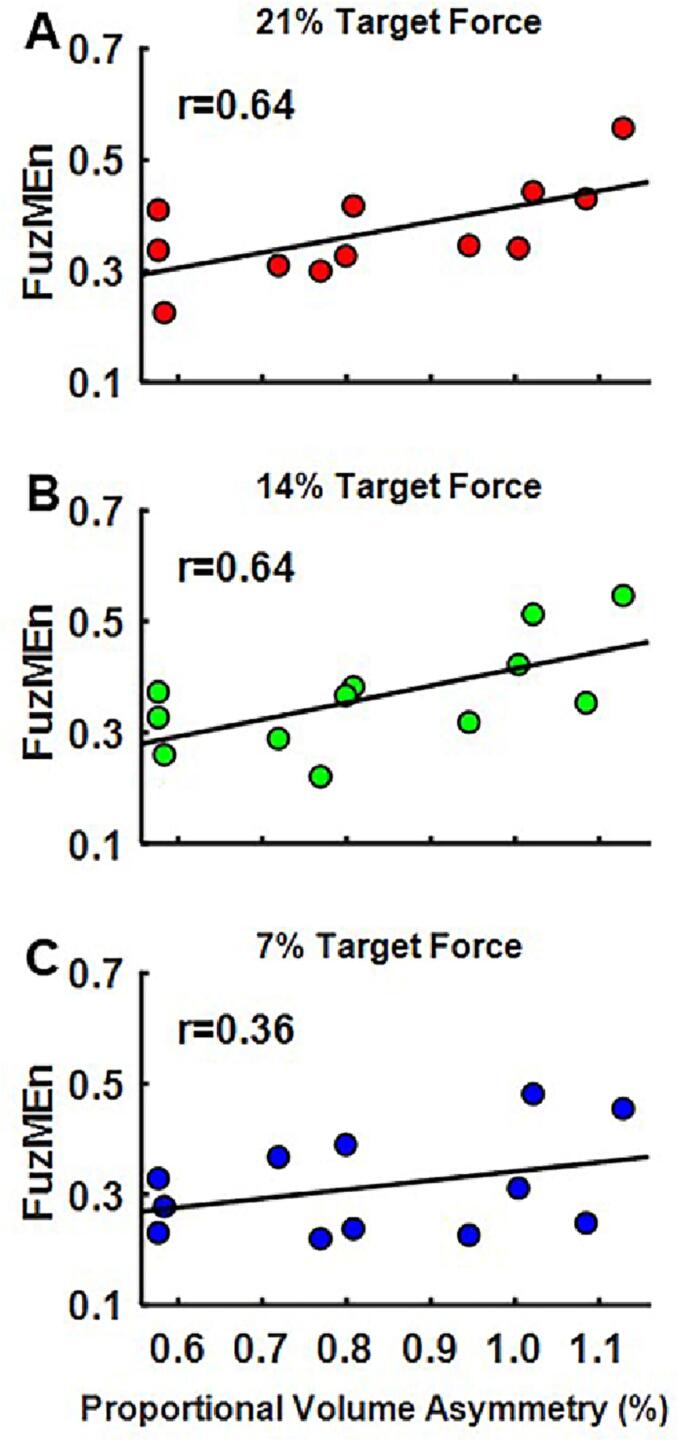
Scatterplots illustrating relation between *proportional volume asymmetry* and *FuzMEn* at A) 7%, B) 14% and C) 21% target force levels in stroke survivors.

The correlation between *proportional volume asymmetry* and *FuzMEn* was not statistically significant at the 7 % force level (*r* = 0.36, *p* = 0.252, Fig. 8A) but reached statistical significance at the 14 % (*r* = 0.64, *p* = 0.025, Fig. 8B) and 21 % (*r* = 0.64, *p* = 0.024, Fig. 8C) force levels. After correcting for the false discovery rate, correlations at 14 % (*p* = 0.036) and 21 % (*p* = 0.036) remained statistically significant.

## 4 Discussion

Findings of the current study show more restricted frequency ranges and reduced temporal irregularity of force oscillations underlying precision grip in humans with longstanding hand impairment secondary to stroke. These features of force output, however, were strongly associated with force-stabilizing ability (i.e., CV and RMSE) in both controls and stroke survivors. Stated another way, force stability was enhanced when spectral power of the force signal was more broadband and temporal irregularity of the time series was increased. Although force output in frequency and time domains tended to modulate with force level, both features were reduced yet uncorrelated with maximal precision grip force in stroke survivors, suggesting that the attenuation was not due to impairments in force-generating capacity. Stroke survivors’ ability to modulate force oscillations at higher frequencies correlated with an asymmetry in the proportional volumes of white matter contained within infarct and mirror compartments of residual and intact CSTs. Collectively, our findings suggest that force stability during precision grip is directly tied to the ability to modulate the frequency content and temporal irregularity of force output, which is altered by stroke and may be explained, at least in part, by residual white matter volume within the descending pathway principally responsible for voluntary distal limb control.

Common motor control deficits after stroke include increased variability and reduced accuracy of the forces produced by the paretic limb, as well as asymmetrical time lags and force magnitudes between the paretic and non-paretic limbs (Kang and Cauraugh, 2015). Stroke survivors in the current study exhibited decreased force-generating capacity and a compromised ability to stabilize precision grip. However, force-generating capacity was not related to force-stabilizing ability across the two higher force levels, possibly because of the low-level forces required by the task paradigm. Stated another way, the ability to transition between stable states at low-level forces is not necessarily due to weakness and can present as equally challenging for survivors with subtler deficits that are more related to higher-order planning. The finding that maximal precision grip force was only correlated with variability about the 7 % target force and not higher force levels might be because the 7 % target force approximates the minimal force needed to maintain grip on the sensor in highly-impaired individuals with low MVCs, making it difficult to keep force at the target level. This interpretation appears consistent with the idea that compromised dexterity due to stroke is not only related to force generation but also force release (Plantin et al, 2022), yet it has been shown that aspects of release are not correlated with MVC (Lindberg et al., 2012). Further work is needed for verification. Another possible influential factor in this regard, which reflects a limitation of the current study, is that precision grip MVC was not normalized between paretic and non-paretic sides.

The frequency composition and temporal irregularity of the force signal was also not associated with force-generating capacity, suggesting that these features are not necessarily due to weakness brought about by the inability to activate lower motor neuron pools. This finding is consistent with a prior study that examined power spectra of paretic knee-extensor isometric contractions across a range of target force levels in individuals within 1 month of stroke onset (Chow and Stokic, 2013). Specifically, lower extremity motor and sensory scores on the Fugl-Meyer assessment did not correlate with frequency content in the force signal. This clinical measure evaluates the integrity of reflexes, as well as single- and multi-joint movements that are rated on an ordinal scale, providing a composite index of limb function. Our findings taken together with this prior work may indicate that oscillations of the force signal provide insight into neural adaptations occurring after injury that are unrelated to gross muscle activation and/or limb function.

Results of the current study also highlight the relevance of force oscillations above 1 Hz, which have been excluded in most prior work (Lodha and Christou, 2017). Findings from previous studies demonstrate that ∼80 % of the change in CV between experimental conditions can be explained by a change in power at low frequencies (Lodha et al., 2013, Moon et al., 2014). In the current study, force stability was predicted (on a target-by-target basis) by a more expansive range of the power spectrum. The frequency composition of force output has been probed in a variety of movement tasks involving upper and lower limbs and across a range of force levels. Only one study to our knowledge has quantified frequency content during submaximal power grip (Lodha et al., 2013) which, for the purpose of comparison, most closely approximates the precision grip task used in experiments described here. Although the task in the prior study involved blocks of trials at each target force level (i.e., 5, 25, and 50 % of MVC) which is in contrast to the pseudo randomization of target force levels used here, both studies show a second discrete peak around 4–7 Hz in the normalized power spectrum averaged across target force levels (see Fig. 3 in Lodha et al., 2013). Low-frequency oscillations have been postulated to decrease force precision, but the effect of manipulating visual feedback (Baweja et al., 2010, Fox et al., 2013) has led to the view that not all oscillations below 1 Hz are detrimental in this regard (Lodha and Christou, 2017). Further support comes from an interventional study that paired upper limb motor retraining with neuromuscular stimulation in stroke survivors (Kang and Cauraugh, 2014). The intervention improved force-stabilizing ability, which was associated with a shift in relative power from a lower-frequency band (0.09–0.41 Hz) to a higher-frequency band (0.59–1.08 Hz). Despite the emphasis on force oscillations below 1 Hz in these studies, our findings support the physiological and functional relevance of faster-frequency contributions to distal limb control.

Subcortical lesions of the CST have been shown to result in higher-order planning deficits (Raghavan et al., 2006). A growing body of evidence supports that motor function very early after stroke onset (Rajashekar et al., 2020) and several months thereafter (Zhu et al., 2010) is inversely related to lesion involvement with the CST and is a strong predictor of recovered hand dexterity (Pennati et al., 2020). To our knowledge, however, no existing studies have examined functional or structural aspect of CST physiology that underlie the ability to modulate force oscillations at higher frequencies. Our preliminary results suggest that the compartment-normalized ratio of white matter volume between residual and intact CSTs is linked to this ability but only at the higher of the two force levels tested. The relative contribution of rate coding and motor unit recruitment varies across the range of force generation in skeletal muscle, but there is evidence that recruitment is a more significant factor in controlling low-level forces (Milner-Brown et al., 1973). Given that paresis results from a disruption in corticospinal input to lower motor neuron pools (Sathian et al., 2011), an association only at the highest target force levels might be explained by compromised regulation of spinal motor neuron recruitment at the lowest range of force generation.

In conclusion, findings from our experiments support growing evidence that highlight the functional relevance of modulating spectral and temporal features of force output to support limb control. Force generation and dexterity after stroke are thought to be controlled by anatomically and functionally distinct systems (Noskin et al., 2008, Xu et al., 2017, Hadjiosif et al., 2022). Primate (Zaaimi et al., 2012) and human (Choudhury et al., 2019) studies have shown an upregulation of the reticulospinal tract after stroke, despite that heightened activity in this subcortical pathway appears less relevant to recovery early after onset (Hammerbeck et al., 2021). Although subcortical pathways have been implicated in the generation of crude patterns of motor output (Zaaimi et al., 2018), our preliminary findings suggest that modulating force output to stabilize precision grip after stroke may be partially accounted for by residual CST volume. Further work is needed for replication in a larger cohort to better understand the role of hand dominance prior to stroke and subcortical contributions to the regulation of force output underlying limb control.

## CRediT authorship contribution statement

**Charley W. Lafe:** Methodology, Software, Validation, Formal analysis, Investigation, Data curation, Writing – original draft, Visualization. **Fang Liu:** Methodology, Software, Validation, Formal analysis, Data curation, Visualization. **Tyler W. Simpson:** Software, Validation. **Chan Hong Moon:** Methodology, Investigation. **Jennifer L. Collinger:** Writing – review & editing. **George F. Wittenberg:** Resources, Writing – review & editing. **Michael A. Urbin:** Conceptualization, Methodology, Software, Validation, Formal analysis, Resources, Writing – original draft, Visualization, Supervision, Project administration, Funding acquisition.

## Declaration of Competing Interest

The authors declare that they have no known competing financial interests or personal relationships that could have appeared to influence the work reported in this paper.

## Data Availability

Data will be made available on request.
